# Raman scattering from the bulk inactive out–of–plane ${{\bf{B}}}_{{\bf{2}}{\bf{g}}}^{{\bf{1}}}$ mode in few–layer MoTe_2_

**DOI:** 10.1038/s41598-018-35510-4

**Published:** 2018-12-10

**Authors:** M. Grzeszczyk, K. Gołasa, M. R. Molas, K. Nogajewski, M. Zinkiewicz, M. Potemski, A. Wysmołek, A. Babiński

**Affiliations:** 1 Faculty of Physics, University of Warsaw, ul. Pasteura 5, 02-093 Warsaw, Poland; 2 Laboratoire National des Champs Magnétiques Intenses, CNRS-UGA-UPS-INSA, 25, Avenue des Martyrs, 38042 Grenoble, France

## Abstract

We report a study of Raman scattering in few-layer MoTe_2_ focused on high-frequency out-of-plane vibrational modes near 291 cm^−1^ which are associated with the bulk-inactive ${{\rm{B}}}_{2{\rm{g}}}^{1}$ mode. Our temperature-dependent measurements reveal a double peak structure of the feature related to these modes in the Raman scattering spectra of 4- and 5-layer MoTe_2_. In accordance with literature data, the doublet’s lower- and higher-energy components are ascribed to the Raman-active A_1g_/${{\bf{A}}{\boldsymbol{^{\prime} }}}_{{\bf{1}}}$ vibrations involving, respectively, only the inner and surface layers. We demonstrate a strong enhancement of the inner mode’s intensity at low temperature for 1.91 eV and 1.96 eV laser light excitation which suggests a resonant character of the Raman scattering processes probed under such conditions. A resonance of the laser light with a singularity of the electronic density of states at the M point of the MoTe_2_ Brillouin zone is proposed to be responsible for the observed effects.

## Introduction

Almost a decade ago layered transition metal dichalcogenides (TMDs), known in solid-state physics since the late 1960s^1^, were rediscovered as a vast group of materials which in the mono- and few-layer form can complement graphene in the quest for alternatives to electronics based on silicon and other conventional semiconductors. Since publication of seminal works of K. F. Mak *et al*.^2^ and A. Splendiani *et al*.^3^ dedicated to molybdenum disulfide, a huge amount of effort has been put into understanding physical and chemical properties of different members of the TMD family. When subject to a more detailed study, thin-film TMDs turned out to offer a number of fascinating possibilities not only in the field of electronics^4,5^, but also optoelectronics^6^, photovoltaics^7,8^, and thermoelectricity^9^, just to mention a few domains of the utmost importance from the research and development point of view. Moreover, they proved themselves to constitute an interesting platform for fundamental investigations. Indeed, due to specific crystal structure, the energy bands and resulting properties of TMDs, composed of layers of covalently bound metal and chalcogen atoms which are kept together by relatively weak van der Waals forces, can be conveniently tuned by simply changing the number of layers (*N*). This is how, in particular, the band gap of semiconducting TMDs is transformed from indirect in the three-dimensional crystals to direct in the monolayer limit. Along with light emission, a strong thickness dependence in TMD materials is exhibited also by their lattice dynamics, commonly probed with the use of Raman scattering (RS) spectroscopy^10^. A pronounced influence of the number of layers on the RS spectrum is observed for both low-energy shear and breathing modes^11,12^ and higher-energy modes of in- and out-of-plane oscillations^13–16^. The impact of sample’s thickness on the lattice vibrations becomes even stronger when resonant excitation of RS is employed. Under such conditions, the resulting change in the electronic configuration of investigated TMD material leads to several effects which cannot be studied with non-resonant RS measurements, such as the enhancement of particular features in the RS spectrum^17^ related to multiphonon processes^18^ or quenching of Raman-active modes due to quantum interference^19,20^. The observation of Davydov splitting of phonon modes in few-layer TMD films, which arises from interlayer interactions, is also facilitated by resonant excitation of RS spectra^21,22^. It has been reported for all semiconducting few-layer TMD materials: MoS_2_^23^, MoSe_2_^24^, WS_2_^25,26^, WSe_2_^27^ and MoTe_2_^28–30^. Last but not least, resonant RS can also unveil those vibrational modes which are symmetry allowed, but barely visible under non-resonant excitation. A prominent example of such "brightening" is the observation of high-frequency out-of-plane modes of A_1g_/${{\rm{A}}^{\prime} }_{1}$ symmetry, which in *N*-layer TMDs originate from the bulk-inactive ${{\rm{B}}}_{2{\rm{g}}}^{1}$ mode^28,31–33^. Surprisingly, in contrast to extensively studied low- and mid-frequency oscillations of few-layer TMD crystals, these modes have not received so far much attention. Strong enhancement of this phonon peak was first reported by Yamamoto *et al*.^31^, where the appearnce in the RS spectrum was atributed to translation symmetry breaking in thin MoTe_2_ layers. The work of Froehlicher *et al*.^28^ exposed the fine doublet structure of these out-of-plane modes at room temperature, which occurs due to surface effects. However, the splitting presented in previous works was rather weak and the additional features were not very distinct. This suggests the sensitivity of the studied system to the resonant conditions is high and should be appropriately adjusted for specific vibrations.

To this aim we employ various excitation wavelengths to measure the RS in few-layer MoTe_2_ and we follow temperature evolution of the RS spectra in 2- to 5-layer-thick MoTe_2_ studied for 1.91 eV and 1.96 eV laser light excitation. A doublet structure of the out-of-plane mode in question, which can be hardly noticed at room temperature, becomes clearly observable at low temperature in tetra- and pentalayer samples. This results from a substantial increase of its lower-energy component’s intensity with decreasing temperature. The experimental results are discussed with respect to a previously reported temperature evolution of the A_1g_/${{\rm{A}}^{\prime} }_{1}$ mode in MoTe_2_^19^. We conclude that the observed temperature evolution of the investigated out-of-plane mode is related to resonant character of the RS for the employed light excitation. We propose that the resonance of the laser light with a singularity of the electronic density of states at the *M* point of the Brilloiun zone in MoTe_2_ might be responsible for the observed anomalous increase of the RS features.

## Results

**Figure 1:**
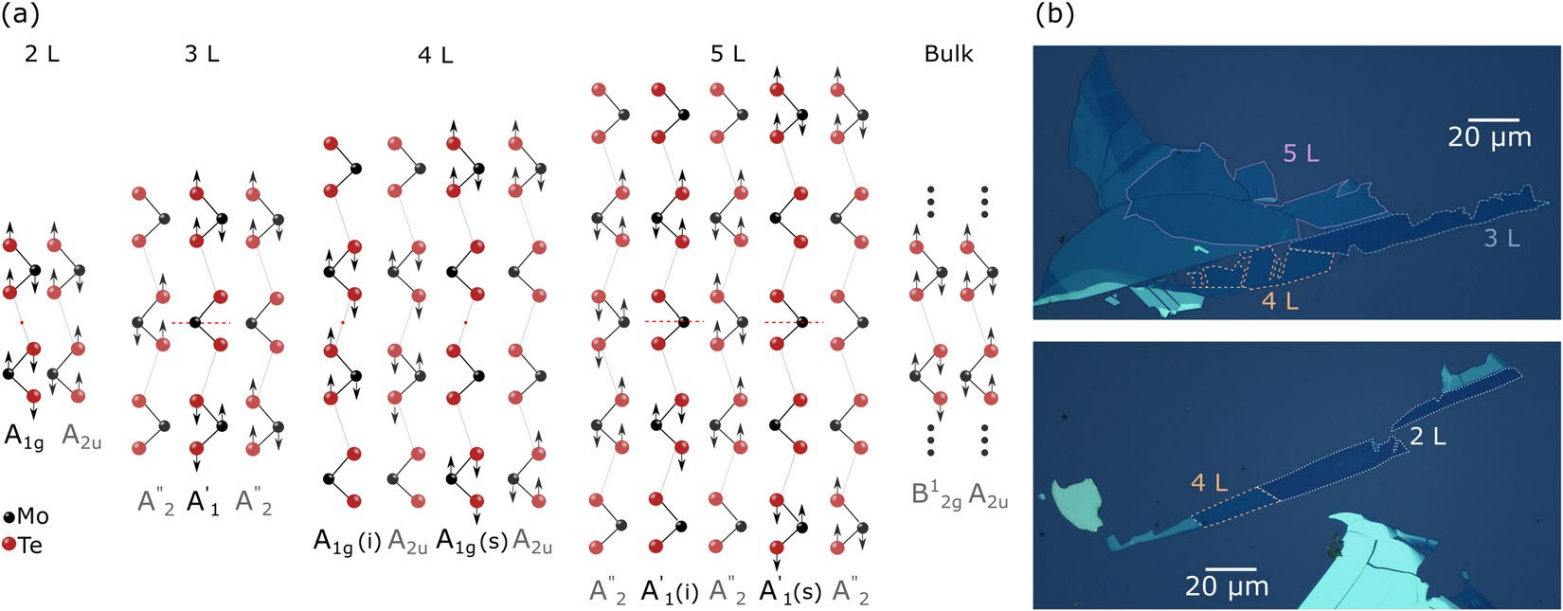
(**a**) Schematic representation of vibrational modes in 2–5 L MoTe_2_. The corresponding bulk-inactive ${{\rm{B}}}_{2{\rm{g}}}^{1}$ mode is also shown for comparison. Dashed red lines denote the mirror symmetry plane and the red points mark the inversion centers. (**b**) Microscopic images of the studied MoTe_2_ flakes.

**Figure 2:**
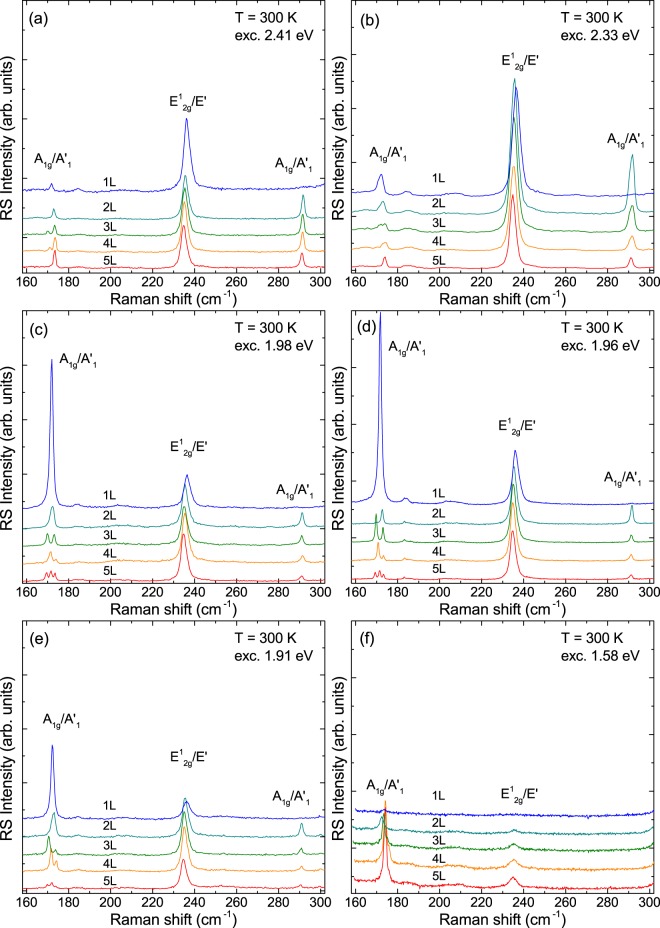
Raman scattering spectra of monolayer (1 L) to pentalayer (5 L) MoTe_2_ measured at room temperature using various excitation energies.

Molybdenum ditelluride (MoTe_2_) is a semiconducting TMD with a relatively small band-gap energy close to 1.3 eV^34^ which makes it well-suited for resonant RS study with the use of commonly available light sources like He-Ne (1.96 eV) or Kr-Ar (1.91 eV) lasers. The former laser energy coincides, in particular, with substantial density of states in few-layer MoTe_2_, which occurs at the *M* and *K* points of the Brillouin zone^32^. Similar to other semiconducting tungsten- and molybdenum-based TMDs, MoTe_2_ crystallizes in a trigonal prismatic (2H) structure, which symmetries in the limit of bulk material belong to the D_6*h*_ point group. As a result, with six atoms in the unit cell, multilayer MoTe_2_ possesses 18 modes of normal vibrations out of which 7 are Raman-active (doubly degenerate in-plane: E_1g_, ${{\rm{E}}}_{2{\rm{g}}}^{1}$ and ${{\rm{E}}}_{2{\rm{g}}}^{1}$, and non-degenerate out-of-plane A_1g_), 6 are infrared (IR)-active (doubly degenerate in-plane ${{\rm{E}}}_{1{\rm{u}}}^{1}$ and ${{\rm{E}}}_{1{\rm{u}}}^{2}$, and non-degenerate out-of-plane ${{\rm{A}}}_{2{\rm{u}}}^{1}$ and ${{\rm{A}}}_{2{\rm{u}}}^{2}$), and 5 are optically inactive (doubly degenerate in-plane E_2u_, and non-degenerate out-of-plane: B_1g_, ${{\rm{B}}}_{2{\rm{g}}}^{1}$ and B_1u_)^35^. On the other hand, the point groups describing symmetries of few-layer MoTe_2_ composed of even and odd number of layers are D_3*d*_ and D_3*h*_, respectively. This leads to different symmetry labelling of vibrational modes depending on the parity of *N*. In a structure with odd (even) *N*, the Raman-active modes are denoted by E′, E″ and ${{\rm{A}}^{\prime} }_{1}$ (E_g_ and A_1g_). The difference in the labelling is presented in Fig. 1(a) for the group of the out-of-plane ${{\rm{B}}}_{2{\rm{g}}}^{1}$-related modes. Figure 1(b) shows microscopic images of the thin MoTe_2_ layers under study. All phonon modes observed in the RS spectra of few-layer MoTe_2_ are presented in Fig. 2, which demonstrates the results obtained for monolayer (1 L) to pentalayer (5 L) excited with several different laser wavelenghts. It can be seen that both the relative intensities and spectral lineshapes of individual peaks for a particular thickness strongly depend on the excitation energy.

Let us focus first on the peaks due to the out-of-plane A_1g_/${{\rm{A}}^{\prime} }_{1}$ vibrations at ~174 cm^−1^, which correspond to the Raman-active A_1g_ mode in bulk MoTe_2_^28–30^. Their characteristic pattern originating from the Davydov splitting can be clearly observed in 3 L, 4 L and 5 L MoTe_2_ for 1.98 eV, 1.96 eV or 1.91 eV, while it remains hardly distinguishable for 1.58 eV excitation (see Fig. 2). The relative intensities of the lower- and higher-energy components of the ${{\rm{A}}^{\prime} }_{1}$ mode in 3 L MoTe_2_ also depend on the excitation energy. The higher-energy component of the ${{\rm{A}}^{\prime} }_{1}$ mode in 3 L MoTe_2_, which is due to vibrations occuring with the same phase in all three covalently bound MoTe_2_ layers is of higher intensity than its lower-energy counterpart in the RS spectrum excited with 2.41 eV light. On the contrary, the lower-energy component due to the mode in which the tellurium atoms in the middle MoTe_2_ layer vibrate out-of-phase as compared to vibrations in the outer layers, is of higher intensity in the RS spectrum excited with 1.96 eV or 1.91 eV light. Finally, the Davydov-split components of the ${{\rm{A}}^{\prime} }_{1}$ mode in 3 L MoTe_2_ are of similar intensity in the RS spectrum excited with 1.98 eV light. As compared to the in-plane mode, the relative intensities of spectral features originating from the out-of-plane mode also strongly depend on the excitation energy. For 1 L MoTe_2_ the out-of-plane mode dominates the RS spectrum excited with 1.98 eV, 1.96 eV or 1.91 eV light, whereas the A_1g_/${{\rm{A}}^{\prime} }_{1}$ peak is much weaker than the one due to in-plane vibrations corresponding to the ${{\rm{E}}}_{2{\rm{g}}}^{1}$ mode in the bulk (observed at ~235 cm^−1^). For 5 L MoTe_2_ the out-of-plane mode is weaker than its in-plane counterpart for 2.41 eV and 2.33 eV excitation while the opposite happens when 1.58 eV excitation is employed. This behaviour indicates a resonant RS of light on the out-of-plane modes, which occurs when the laser energy coincides with a maximum of electronic density of states in the crystal lattice^32^. This effect was previously reported for MoTe_2_^29,30,36^ and explained in terms of quantum interference of contrubutions from different points of the Brillouin zone and the electron-phonon coupling^20^. A possible role of resonant vs non-resonant contributions to the RS efficency was also suggested in ref.^19^.

**Figure 3:**
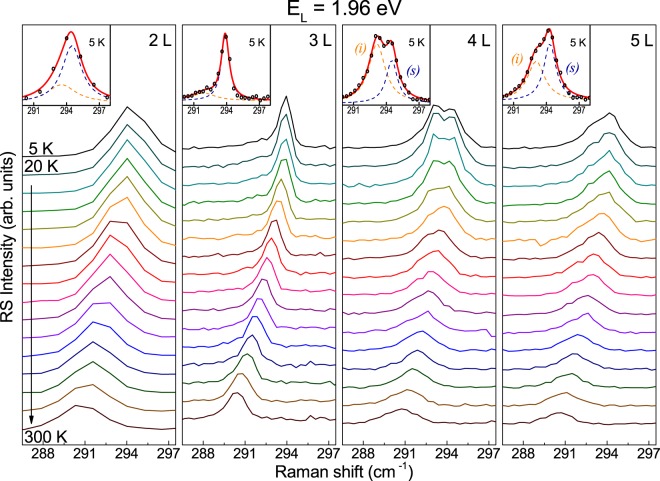
Temperature evolution of high-energy part of the Raman scattering spectra of 2–5 L MoTe_2_ with the out-of-plane A_1g_/${{\rm{A}}^{\prime} }_{1}$ modes corresponding to the bulk inactive ${{\rm{B}}}_{2{\rm{g}}}^{1}$ mode. Laser excitation energy equals 1.96 eV (*λ* = 632.8 nm).

**Figure 4:**
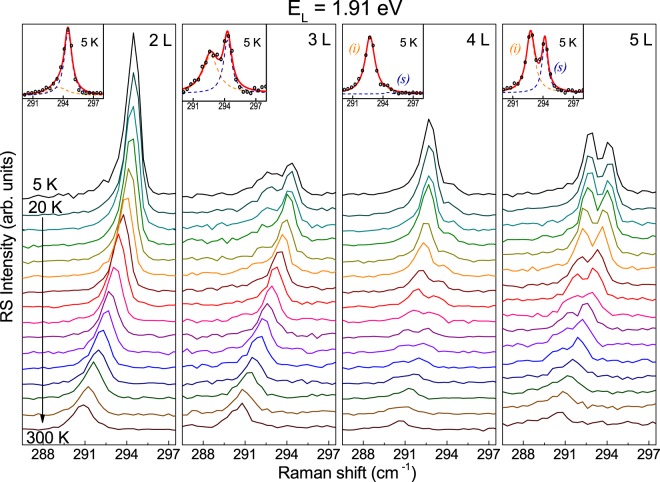
Temperature evolution of high-energy part of the Raman scattering spectra of 2–5 L MoTe_2_ with the out-of-plane A_1g_/${{\rm{A}}^{\prime} }_{1}$ modes corresponding to the bulk inactive ${{\rm{B}}}_{2\text{g}}^{1}$ mode. Laser excitation energy equals 1.91 eV (*λ* = 647.1 nm).

A focal point of this paper is the feature apparent at ~291 cm^−1^ in the RS spectra of flakes composed of at least two MoTe_2_ layers. Because of symmetries identifing this mode it is labelled as A_1g_/${{\rm{A}}^{\prime} }_{1}$ for few-layer thick structures. The Mulliken notation is exactly the same as for the peaks at ~174 cm^−1^ described in the preceding paragraph. Therefore, in order to avoid any confusion, unless otherwise stated, the A_1g_/${{\rm{A}}^{\prime} }_{1}$ label will always refer in what follows to the feature at ~291 cm^−1^. It corresponds to vibrations in which the tellurium atoms in each layer oscillate out-of-phase with respect to the molybdenum atoms (see Fig. 1). There are two such modes in bulk MoTe_2_. The atoms in neighboring layers vibrate out-of-phase in the optically inactive ${{\rm{B}}}_{2\text{g}}^{1}$ mode while in the infrared-active (A_2u_) mode the in-phase vibrations occur. As a result none of the modes contribute to the RS in bulk MoTe_2_. Raman-inactive (IR-active) is also the corresponding ${{\rm{A}}}_{2}^{^{\prime\prime} }$ mode in monolayer MoTe_2_. Additional symmetry elements, like the inversion symmetry center for even or the mirror plane for odd number of layers, activate the phonon mode in the material consisting of at least two MoTe_2_ layers. Two out-of-plane modes in 2 L MoTe_2_ are similar to those present in the bulk. However, due to the symmetry, the A_1g_ mode, in which the atoms in both layers move out-of-phase with respect to each other becomes Raman-active (see Fig. 1). The other mode, A_2u_, in which the vibrations in both layers occur in-phase, is IR-active. A similar line of reasoning may be applied to any other *N*-layer structure in which *N* is an even number. The number of Raman-active A_1g_ modes and the number of IR-active A_2u_ modes in such cases equals $\frac{N}{2}$^28^. If *N* has an odd value, the Raman-active modes must hold the mirror symmetry with respect to the plane crossing the Mo atoms in the central layer (see Fig. 1). The number of these ${{\rm{A}}^{\prime} }_{1}$ modes is equal to $\frac{N-1}{2}$ and the number of corresponding IR-active ${{\rm{A}}}_{2}^{^{\prime\prime} }$ modes amounts to $\frac{N+1}{2}$^28^. The above considerations lead, in particular, to a conclusion that there are: one Raman-active ${{\rm{A}}^{\prime} }_{1}$ mode and two ${{\rm{A}}}_{2}^{^{\prime\prime} }$ IR-active modes in 3 L MoTe_2_, two Raman-active A_1g_ modes and two A_2u_ IR-active modes in 4 L MoTe_2_, and two Raman-active ${{\rm{A}}^{\prime} }_{1}$ modes and three IR-active ${{\rm{A}}}_{2}^{^{\prime\prime} }$ modes in 5 L MoTe_2_. Corresponding normal mode displacements are schematically presented in Fig. 1. Two different modes of Raman-active vibrations in 4 L and 5 L samples are referred to as the surface mode (s) and inner mode (i)^28,33^. The atomic displacements in the corresponding normal modes occur in the outside layers (s) and in central layers (i), respectively. Based on theoretical calculations^28^ the energy splitting between the modes has been estimated to be ~1.2 cm^−1^. Previous works attributed the splitting mainly to surface effects. The Davydov splitting, which is due to van der Waals interactions between neighboring layers (being of the order of ~0.1 cm^−1^) is negligible in this case. Experimental study carried out using 1.96 eV excitation show that the inner mode appears in the RS spectra of *N* ≥ 4 L MoTe_2_ as a faint shoulder on the peak related to the surface mode^28^. Our results confirm that observation. However, the lower-energy peak related to the bulk inactive ${{\rm{B}}}_{2{\rm{g}}}^{1}$ mode can be observed in the spectra measured at room temperature just as a weak feature (see Fig. 3). In order to improve its visibility by changing the excitation conditions, we have studied the RS as a function of temperature. It is well known that the semiconductor energy band structure changes with temperature with the band gap usually widening when the temperature is lowered. Temperature modulation therefore allows to form variable resonance conditions without changing the laser excitation energy^17,19^. The effect of temperature on the investigated RS can be appreciated in Figs 3 and 4 showing a series of RS spectra excited with 1.96 eV and 1.91 eV light and measured as a function of temperature ranging from 5 K to 300 K on 2–5 L MoTe_2_.

It can be clearly seen in Figs 3 and 4 that the lineshape of the investigated spectral features substantially changes with decreasing temperature. Let us start the analysis of the results with the most staggering effects observed for 4 L and 5 L MoTe_2_. For both excitation energies, the lower-energy component of the doublet significantly gains the intensity with decreasing temperature. The effect can be appreciated for both MoTe_2_ structures, however, at *T* = 5 K it is stronger for 4 L than for 5 L flakes. It is also stronger for 1.91 eV than for 1.96 eV excitation. At *T* = 5 K the lower-energy component of the A_1g_/${{\rm{A}}^{\prime} }_{1}$ modes dominates the high-energy part of the RS spectrum excited with 1.91 eV. Following the theoretical disscusion presented in ref.^28^ we attribute the higher-energy component of the investigated peak in 4 L and 5 L to the surface mode (s) of the out-of-plane A_1g_ or ${{\rm{A}}^{\prime} }_{1}$ vibrations, respectively, which involve atomic dispacements in the outer MoTe_2_ layers (see Fig. 1). The lower-energy component is the Raman-active A_1g_/${{\rm{A}}^{\prime} }_{1}$ inner mode (i) in 4 L/5 L MoTe_2_. The energy splitting between the components matches the value predicted by a linear chain model. This points at surface effects as the main factors which affect the splitting between the surface and the inner mode in the sample. An observation, which goes beyond the previously reported data, concerns the full width at half maximum (FWHM) of both components (see Table 1).

**Table 1:** Full width at half maximum (FWHM) of individual contributions to the A_1g_/${{\rm{A}}^{\prime} }_{1}$ features in the RS spectra collected at *T* = 5 K, obtained by fitting the experimental data with a superposition of two Lorentzian peaks.

Excitation energy (eV)	FWHM (cm^−1^)							
	2 L		3 L		4 L		5 L	
	A_2u_	A_1g_	${{\rm{A}}}_{2}^{^{\prime\prime} }$	${{\rm{A}}^{\prime} }_{1}$	A_1g_(i)	A_1g_ (s)	${{\rm{A}}^{\prime} }_{1}$(i)	${{\rm{A}}^{\prime} }_{1}$ (s)
1.96	3.2	2.2	2.6	0.9	1.8	1.2	1.9	1.3
1.91	2.2	0.9	1.6	0.9	1.2	0.5	1.0	0.7

The lower-energy components of the investigated doublets are broader than their higher-energy counterparts. This may suggest that the former features correspond to more than one vibrational mode. Additional vibrations which may contribute to the lower energy peak are most likely the IR-active modes. They are expected to be quasi-resonant with the inner modes. Although they should not appear in the RS spectrum, it is known that resonant excitation and/or disorder in the structure can make them Raman-active^25,33,37^. This conclusion is supported by the RS from 2 L and 3 L MoTe_2_ (see Figs 3 and 4). In both cases there is just one Raman-active mode of the out-of-plane vibrations, which is actually the surface mode. However, at low temperature, the lower-energy peak related to the out-of-plane vibrations becomes apparent in the spectrum. Its intensity is lower than the intensity of its higher-energy counterpart but its presence in the RS spectra remains beyond any doubts. As in the case of 4 L and 5 L MoTe_2_, also for 2 L and 3 L MoTe_2_ the peaks can be seen more distinctively for 1.91 eV excitation.

**Figure 5:**
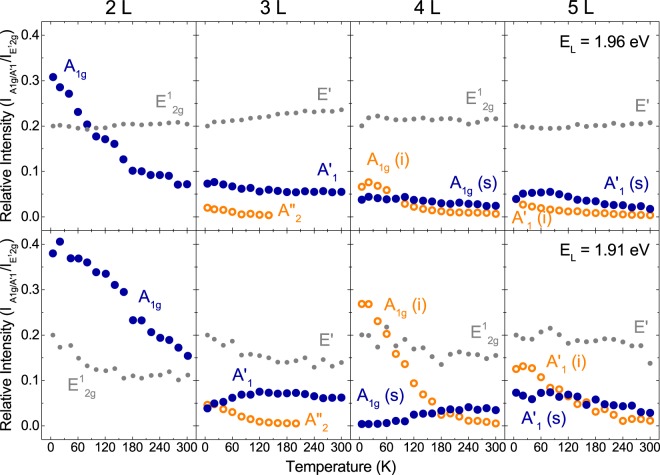
Temperature dependence of the relative intensities of the A_1g_/${{\rm{A}}^{\prime} }_{1}$ - related peaks in the Raman scattering spectra of 2–5 L MoTe_2_ excited with 1.96 eV (*λ* = 632.8 nm) - top panel and 1.91 eV (*λ* = 647.1 nm) laser light energy - bottom panel. The grey points present the corresponding dependence of the ${{\rm{E}}}_{2{\rm{g}}}^{1}$/E′ modes intensities, normalized to intensity of the mode ${{\rm{E}}}_{2{\rm{g}}}^{1}$/E′ at 5 K and mulitplied by 0.2 to roughly match values of other data.

In order to highlight the resonant character of changes in the RS spectra of few-layer MoTe_2_ described above, the best way is to plot the intensities of individual spectral features as a function of temperature. For better comparison, it is convenient to relate the intensities of the out-of-plane modes to the intensity of the in-plane E_g_/E′ mode, as this mode is not expected to exhibit any resonant behaviour (see the grey points in Fig. 5). Such an approach accounts for small variations of the excitation laser intensity^17^.

The temperature dependences of the relative intensities of the A_1g_/${{\rm{A}}^{\prime} }_{1}$ related peaks observed in the RS spectra of 2–5 L MoTe_2_ samples under study are presented in Fig. 5. As previously stated, the most pronunced is the effect of temperature on the RS spectrum excited with 1.91 eV light. The peak due to the A_1g_ (s) mode in 4 L MoTe_2_ looses its intensity with decreasing temperature. This behaviour is opposite to the temperature evolution of the peak related to the A_1g_ (i) mode (with a possible contribution from the IR-active out-of-plane mode). This peak is almost seven times more intense at *T* = 5 K than at room temperature. A similar effect of temperature can be noticed for spectral features of 5 L MoTe_2_. In this case, the ${{\rm{A}}^{\prime} }_{1}$ (i) mode is three times stronger at *T* = 5 K than at room temperature. We interpret the observed effect of temperature on the out-of-plane vibrational modes in terms of the transformation of the MoTe_2_ band structure induced by changing the temperature, which we discuss in more detail in the following section.

## Discussion

It has previously been noted that the resonance of the exciting light with the electronic transitions in a semiconductor structure can result in strong enhancement of particular RS modes^38^. This effect was observed in several semiconductor TMDs including bulk MoS_2_^17^. It was shown that modification of the MoS_2_ band structure caused by either the temperature or pressure can lead to significant increase of the intensity of the RS peak due to the out-of-plane Raman-active A_1g_ mode. We propose a similar explanation for the observed enhancement of the A_1g_ (i) mode in 4 L MoTe_2_ reported in this work.

In our case, however, a two-dimensional space of tuning parameters which affect the MoTe_2_ band structure is spanned by temperature (*T*) and the number of layers in the sample (*N*). Symbolically, it can be expressed by a formula *E*_*i*_ (***k***, *T*, *N*), where *E* stands for the energy, *i* is the band index, and ***k*** indicates the wave vector. Following this formalism we can say that the results shown in the bottom panel of Fig. 5 clearly demonstrate that the excitation energy of 1.91 eV employed in our experiment corresponds to such a point in the *E*_*i*_ (***k***, *T*, *N*) space, where in the vicinity of a particular ***k***, *T* = 5 K and *N* = 4 a substantial density of electronic states exists in the MoTe_2_ bands linked by optically active transitions. As also shown in Fig. 5, tuning *E, N*, or *T* by as little as 50 meV, plus/minus one layer, and 100 K, respectively, while keeping the other two parameters constant, can efficiently quench the intensity of the A_1g_-mode-related peak in the RS spectrum of few-layer MoTe_2_. It shows how sensitive the resonant RS is as a tool to probe the local density of electronic states in a semiconductor system.

We suggest that the strong enhancement of the A_1g_(i) mode’s intensity in the RS spectrum of 4 L MoTe_2_ is associated with electronic transitions taking place at the so-called *M* point of the Brillouin zone of the hexagonal crystal lattice. Indeed, as demonstrated by transmission measurements performed on bulk^1,39^ and few-layer 2H-MoTe_2_^30,34^, in the vicinity of 1.91 eV there exists a substantial density of electronic states which gives rise to a doublet of excitonic transitions known as A′ and B′. Their labelling intentionally resembles that of the optical transitions at the *K* points of the Brillouin zone as in both cases the local minima and maxima of the conduction and valence band, respectively, are split into pairs of subbands by spin-orbit interaction. Essentially independent of the sample thickness, the A′ transition occurs at room temperature at about 1.73 eV whereas the B′ is observed at about 1.98 eV. With decreasing temperature, due to thermal shrinkage of the crystal lattice and resulting effective repulsion of the energy bands, the energies of all excitonic transitions experience a blueshift. For the lowest-energy *K*-point transition between the spin-orbit-split subbands in the conduction and valence bands of 1 L MoTe_2_ it amounts to about 0.1 eV as inferred from the comparison of optical data recorded at 4.5 K^34^ and room temperature^40^. Worth noting here is that the blueshift of fundamental band-gap transitions is about 0.1 eV when going from room to liquid helium temperature is characteristic of all monolayer TMDs, as shown by temperature-dependent reflectance contrast measurements performed on WSe_2_^41^, MoSe_2_^42^, and WS_2_^43^. To the first approximation, the same temperature evolution as for the *K* points can be assumed also for transitions taking place at other points of the Brillouin zone, as the uniform change in the equilibrium distance between atoms in the MoTe_2_ crystal lattice should have a homogeneous impact on the whole band structure. We should then expect the A′ and B′ transitions to occur at 4.5 K at about 1.83 eV and 2.08 eV. Although it is difficult to precisely estimate the broadening of these transitions based on the experimental results, presented in refs^1,30,34,39^, it must amount to at least a few tens of meV. It means that with 1.91 eV excitation we most probably probed the high-energy shoulder of the maximum in the local density of electronic states which gives rise to the A′ transition. This conjecture also explains the sensitivity of our results to the excitation energy since changes in the resonance conditions should be much more dynamic for the slope than for the close-to-maximum part of the peak in the local density of states. In this respect it would be desirable to extend our study to excitation energies falling in the range from about 1.75 eV to 1.91 eV.

A question arises why only the inner modes are in resonance with 1.91 eV laser excitation, while the surface modes react to it relatively weakly. Definitely, a more formal theoretical approach is necessary to account for this observation. We suppose, however, that it may be related to the effect of particular electronic excitation on the polarizability of bonds involved in different vibrational modes. For example, as it was demonstrated in ref.^20^, for 3 L MoTe_2_ the contributions from different regions of the Brillouin zone add with particular signs (plus or minus). Consequently, this may lead to a partial quenching of some features observed in the RS spectrum. This effect can be easily recognized for the high-energy component of the out-of-plane ${{\rm{A}}^{\prime} }_{1}$ vibrations in MoTe_2_, where the contributions from the vicinity of the *M* point partially cancel each other out for *E*_*L*_ = 1.96 eV excitation^19,20^. This is not the case of the low-energy part of the mode. We believe that a similar effect is responsible for our observations. Temperature modulation of the MoTe_2_ band structure modifies resonant excitation conditions and may result in different effects on individual vibrational modes.

## Conclusions

In conclusion, we have investigated Raman scattering from the out-of-plane vibrational modes (A_1g_/${{\rm{A}}^{\prime} }_{1}$) in few-layer MoTe_2_ of thickness ranging from one to five layers. Our temperature-dependent measurements performed with the use of several excitation energies clearly demonstrate a doublet structure of one of the features corresponding to these modes in the RS spectrum at about 291 cm^−1^, especially pronounced in 4 L- and 5 L-thick samples excited with 1.91 eV and 1.96 eV laser light. The lower- and higher-energy components of the doublet have been identified as the inner and surface modes of the out-of-plane oscillations, respectively. We have shown that for 1.91 eV excitation a strong enhancement of the inner mode’s contribution to the RS spectrum is observed for 4 L and 5 L MoTe_2_. Based on temperature evolution of this effect, we qualitatively interpret it as being associated with the resonance between the A_1g_ (i) mode and the A′ excitonic transition at the *M* point of the MoTe_2_ Brillouin zone, which at 4.5 K is expected to occur at about 1.83 eV. Taking into account the finite broadening of the A′ transition (at the level of few tens of meV), the enhancement of the A_1g_ (i)-mode-related feature’s intensity in the RS spectrum we have demonstrated for 1.91 eV excitation can be understood as originating from the onset of resonant conditions (from the high-energy side) reaching their maximum at slightly lower energies, not yet available in the experimental set-up exploited for the measurements reported in this paper. A striking observation about the resonance mentioned above is its extreme sensitivity to the sample thickness which cannot result from the MoTe_2_ band structure, which at the *M* point of the Brillouin zone barely changes with the number of layers in the sample. It may hint at important details we have not considered in our simplified interpretation, like, for instance, the thickness and temperature dependence of electron-phonon interactions. Definitely, a much more strict and formal analysis of our experimental data is necessary to fully account for all the results reported in this manuscript, not only qualitatively but also at the quantitative level. We do hope that the present work will stimulate a theoretical study which will shed more light on many aspects of resonant Raman scattering in TMD systems which have not yet been thoroughly understood.

## Methods

MoTe_2_ samples were prepared by an all-dry PDMS-based exfoliation technique^44^ of a bulk crystal purchased from HQ graphene and deposited onto a Si/(90 nm) SiO_2_ substrate. The thickness of studied MoTe_2_ flakes was determined by optical contrast and Raman spectroscopy.

The unpolarized RS measurements were carried out in the backscattering geometry. Raman spectra were taken at various laser excitation wavelenghts: 785 nm (1.58 eV), 625.4 nm (1.98 eV), 632.8 nm (1.96 eV), 647.1 nm (1.91 eV), 514.5 nm (2.41 eV), and 532 nm (2.33 eV). The Stokes scattering was measured on 2–5 L MoTe_2_ in a temperature range from 5 K to 300 K. The investigated samples were placed on a cold finger of a continuous flow cryostat, mounted on the x-y motorized positioners. The excitation light was focused by means of a 50x magnification long working distance objective. The spot diameter of the focused beam was 1 μm. The excitation power focused on the sample was kept at 100 μW during all measurments to avoid local heating. The RS signal was collected via the same microscope objective, sent through a 0.75 m monochromator and detected with a liquid-nitrogen cooled CCD camera.

## Data Availability

The datasets obtained during experiments and analysis in course of manuscript preparation are available from the corresponding author on reasonable request.
